# Paracentral Acute Middle Maculopathy Associated With Cilioretinal Artery Insufficiency Following COVID-19 Vaccination in a Young Patient

**DOI:** 10.7759/cureus.28739

**Published:** 2022-09-03

**Authors:** Dimitra Katerini, Myron Z Markakis, Vasiliki Kounali, Konstantina Koulotsiou, Dimitris Dimopoulos, Michail Nodarakis, Andreas Zacharioudakis, Pavlos Koutentakis

**Affiliations:** 1 Department of Ophthalmology, Venizeleio General Hospital of Heraklion, Heraklion, GRC; 2 Department of Ophthalmology, G. Gennimatas General Hospital, Athens, GRC

**Keywords:** cilioretinal artery insufficiency, retina and vaccine, visual acuity measurement, cilioretinal artery, optical coherence tomography, covid-19 vaccine, paracentral acute middle maculopathy

## Abstract

We aim to present a unique case of unilateral paracentral acute middle maculopathy (PAMM) associated with cilioretinal artery insufficiency following the coronavirus disease 2019 (COVID-19) vaccination. A 28-year-old male complained of a sudden blurring of vision in his left eye 40 days after receiving the second dose of COVID-19 immunization. The optical coherence tomography revealed a diffuse paracentral area of hyper-reflective change in the inner plexiform layer and an increase in the inner nuclear layer volume, consistent with PAMM along the course of the cilioretinal artery. PAMM has been connected to an assortment of retinal vasculature anomalies. Considering COVID-19 vaccination, we hypothesize that the immunogenic cascade following vaccination dysregulated coagulation and led to retinal vascular thrombosis. However, the link between COVID-19 vaccination and retinal vascular occlusion disease remains unknown.

## Introduction

Sarraf et al. [1] first described paracentral acute middle maculopathy (PAMM) as a hyper-reflective band-like lesion involving the inner nuclear layer (INL), resulting in permanent INL thinning due to INL atrophy, which is typical of tissue hypoperfusion. It is often associated with an underlying condition causing microvascular ischemia. Herein, cilioretinal artery insufficiency (CILRAI) was present. CILRAI is an uncommon variant of artery pathology. It is defined as a transient hemodynamic block because of the elevated intraluminal capillary pressure [2].

## Case presentation

A 28-year-old male presented to the ophthalmology department with acute, painless vision loss of sudden onset in his left eye. He did not have any additional systemic or ocular problems. He had an unremarkable medical history and was free of medications. His ocular history was only significant for bilateral laser-assisted in situ keratomileusis (LASIK) surgery to treat myopia. The patient had received the second dose of SARS-CoV-2 vaccination (Pfizer-BioNTech coronavirus disease 2019 (COVID-19) vaccine) 40 days earlier.

A comprehensive ophthalmologic examination was carried out on the patient. His presenting best-corrected visual acuity (BCVA) on the Snellen decimal chart was 20/20 in the right eye and counting fingers (CF) in the left. Anterior segment examination revealed no abnormal findings. Bilateral intraocular pressure was 14 mmHg. Eye movements were within normal limits, and no discomfort was felt. Both eyes showed normal pupillary light reflexes.

**Figure 1:**
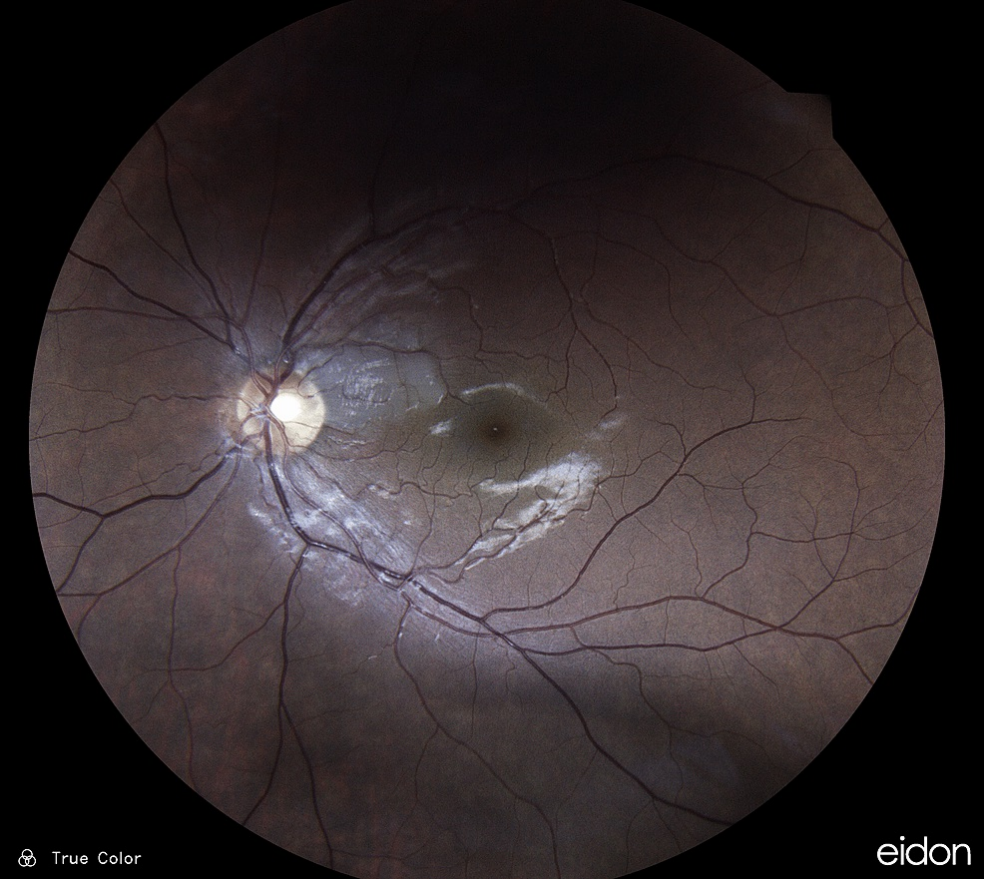
Patient's left fundus upon presentation A dark grey parafoveal wedge-shaped area was present secondary to localized retinal edema on the distribution of a possible cilioretinal artery in the left eye.

Ophthalmoscopic examination of the retina revealed a dark grey parafoveal wedge-shaped area secondary to localized retinal edema on the distribution of a possible cilioretinal artery in the left eye (Figure 1).

**Figure 2:**
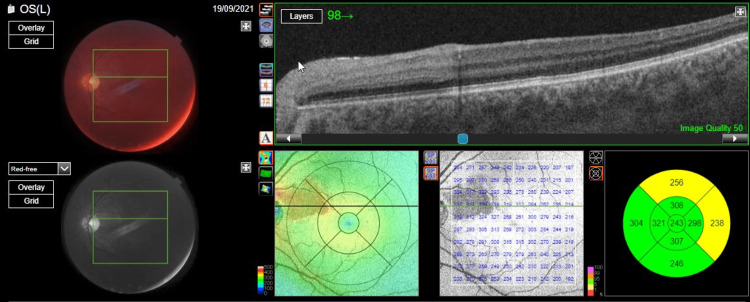
Swept-source optical coherence tomography of the patient's left fundus We can observe a band-like hyper-reflective lesion starting from the inner plexiform layer (IPL) to the outer plexiform layer (OPL) in a paracentral location consistent with paracentral acute middle maculopathy.

No further abnormalities were observed during fundoscopy, and no macular edema was present at that time. Registered swept-source optical coherence tomography (SS-OCT; Topcon, Japan) demonstrated a characteristic band-like hyper-reflective lesion starting from the inner plexiform layer (IPL) to the outer plexiform layer (OPL) in a paracentral location consistent with PAMM (Figure 2).

Amsler grid test was positive, and standard automated perimetry full threshold 80/30 (DICON LD400, Vismed Dicon, San Diego, CA) showed a paracentral scotoma of the left eye, which corresponds to the area of retinal ischemia and respected the horizontal meridian.

The next day, a fundus fluorescein angiography (FFA) was conducted. FFA confirmed the presence of a patent cilioretinal artery.

**Figure 3:**
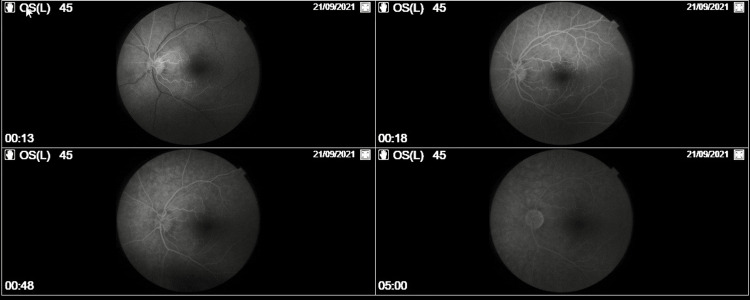
Patient's fundus fluorescein angiography Fundus fluorescein angiography confirmed the presence of a patent cilioretinal artery in the patient's left eye. The flow within the cilioretinal artery was present and confirmed, but the surrounding region was ischemic.

The flow within the cilioretinal artery was present and confirmed, but the surrounding region was ischemic (Figure 3). No signs of inflammation were observed. Nevertheless, FFA cannot clearly distinguish the intermediate capillary plexus (ICP) from the deep capillary plexus (DCP) [3]. PAMM associated with CILRAI was diagnosed.

A full medical evaluation of the patient was performed. Reverse transcription-polymerase chain reaction (RT-PCR) for COVID-19 came back negative. A complete blood count, activated partial thromboplastin time and prothrombin, a lipid profile, and serum glucose levels were all included in the relative workup. Complete cardiac evaluation with arterial blood pressure, electrocardiogram, and heart echo was normal. Additional carotid Doppler ultrasound was used to detect possible stenosis, without detecting any pathology. Laboratory and imaging tests (homocysteine levels and angiotensin-converting enzyme levels) were also normal, ruling out other infections, immunologic, and hypercoagulable states. Furthermore, the hematology department was consulted regarding thrombophilia and anti-phospholipid syndrome, and protein C, protein S, and autoantibodies were normal in the examination. Brain MRI and magnetic resonance angiography revealed no abnormal findings as well. At that time, no indications for intervention were present so the patient was monitored closely.

**Figure 4:**
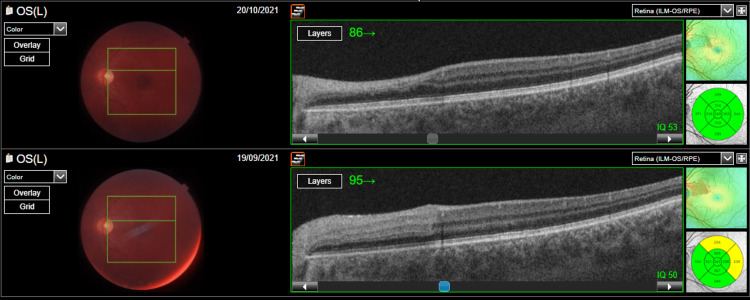
Optical coherence tomography of the patient's left eye upon presentation and two weeks after the event Two weeks after presentation, the initial inner retinal edema area is restored by thinning.

During the two-week follow-up period, visual acuity quickly normalized to 20/20 in the left eye, and on the visual field test, the paracentral depression persisted. Optical coherence tomography (OCT) demonstrated that thinning has replaced the area of intraretinal edema (Figure 4).

**Figure 5:**
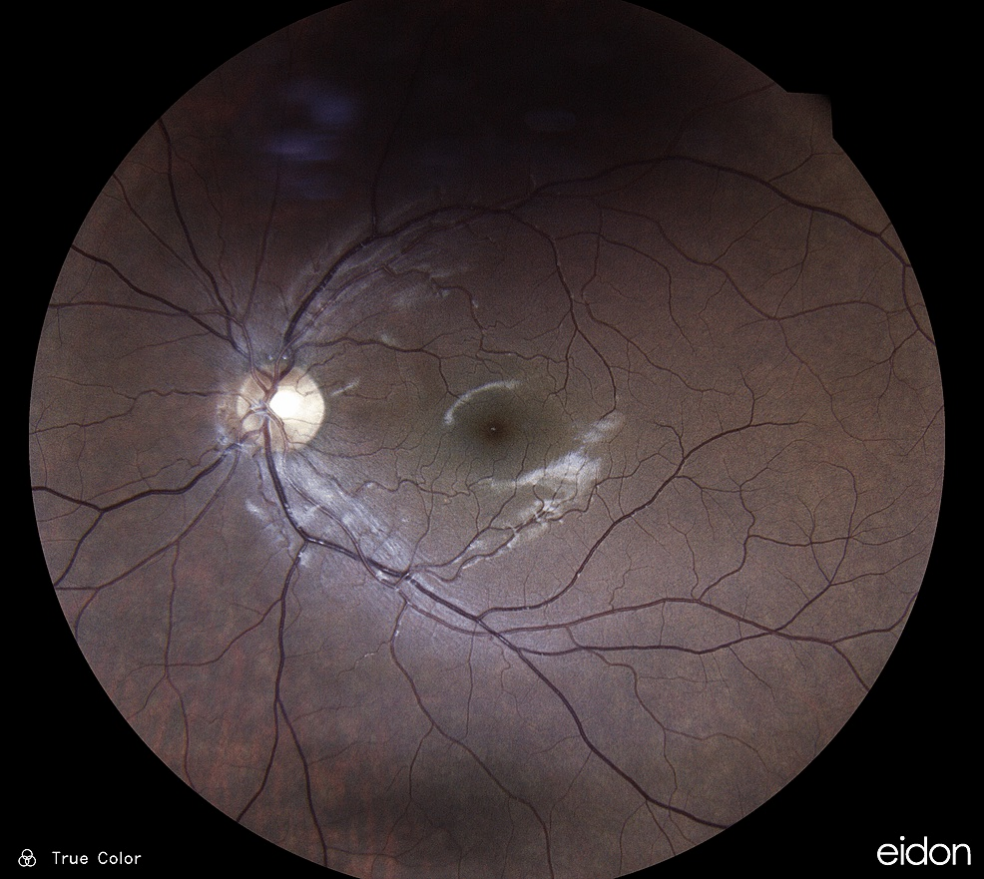
Patient's left fundus in the follow-up, three months after the initial event The fundus findings were mainly diminished.

Furthermore, three months after the onset of symptoms, the depression improved further, but thinning of the inner retinal layers was observed (Figure 5).

## Discussion

PAMM is a newly identified OCT finding characterized by a hyper-reflective band affecting the INL, resulting in INL atrophy that is responsible for persistent visual field depressions [3]. Although its pathophysiology is still unknown, ischemic hypoxia at the ICP and DCP has been shown to play a significant role [4]. OCT angiography studies have revealed changes in the vasculature of the ICP and DCP [5].

PAMM has been linked to a variety of retinal vasculature abnormalities [1], including retinal artery or venous occlusions, cilioretinal artery (CILRA) occlusion, hypertensive retinopathy, sickle cells, and Purtscher retinopathies. The fovea and a small portion of the papillomacular bundle are supplied by the CILRA. In contrast to the central retinal artery, the CILRA lacks defense mechanisms against blood flow fluctuations [4]. Pichi et al. [6] found PAMM lesions in all eyes with isolated cilioretinal artery obstruction (CILRAO), which they linked to hypoperfusion of the deep vascular plexus and an ischemic insult to the middle retinal layers, particularly INL. Also, they discovered that isolated CILRAI is related to ischemic retinal whitening along the route of the artery and PAMM affecting the middle retina, as illustrated by OCT [6]. As a result of its deeper placement in the watershed between the retina and the choroid, INL is more susceptible to low oxygen levels.

Numerous risk factors have been associated with CILRAI, although healthy individuals may also present with CILRAI [7]. Atherosclerosis, thrombophilia, vasculitis, and other autoimmune disorders have also been proposed. Patent foramen ovale, elevated homocysteine levels, drug abuse, and rare infections should all be ruled out in younger people, as shown in this case.

Patients with COVID-19 infection are known to be prone to thrombosis; cases of thrombosis in the retinal arteries and veins have been reported [8]. Vinzamuri et al. [9] described one case of a young patient with bilateral PAMM and acute macular neuroretinopathy post-COVID-19 immunization. Additionally, an association between COVID-19 vaccination (Pfizer-BioNTech vaccine) and retinal vein occlusion was reported by Tanaka et al. [10], further contributing to the hypothesis that the immunogenic cascade after vaccination dysregulated coagulation and leads to thrombosis of the retinal vascular system.

PAMM cases have an excellent visual prognosis with good final visual acuity, as PAMM represents the earliest form of retinal infarction [4]. When associated with CILRAI, the prognosis is varied and largely depends on the duration and severity of retinal ischemia from the CILRA [7]. Here, flow within the artery was normalized rapidly, as the FFA verified.

## Conclusions

PAMM has been associated with deep vascular complex ischemia, and because cilioretinal arteries are final arteries, their insufficiency may result in regional infarction of the INL. Although absolute certainty is hard to establish, we feel that the association between CILRAI and COVID-19 vaccination is feasible. COVID-19 immunization is justified as a necessary public health intervention, and all approved vaccinations have been shown to be both safe and efficacious. Our case report adds to other reports of potential side effects and post-immunization complications that ophthalmologists should be aware of.
